# Banking on the Neighborhood? Examining the Role of Proximity to Local Banking in Older Adults’ Well-Being

**DOI:** 10.1093/geroni/igaf122.1410

**Published:** 2025-12-31

**Authors:** Alyssa Goldman, Megan Bea

**Affiliations:** Boston College, Chestnut Hill, Massachusetts, United States; University of Wisconsin–Madison, Madison, Wisconsin, United States

## Abstract

Access to local banking represents an understudied dimension of neighborhood-based inequalities that could significantly influence older adults’ perceptions of their neighborhood spaces in ways that matter for disparities in well-being. Building on a 2024 publication by the authors, we evaluate disparities in banking access and then examine how local banking access informs older adults’ perceptions of their neighborhood, which hold important implications for well-being. We use nationally representative data from older adults in the United States who were interviewed at Round 3 of the National Social Life, Health, and Aging Project, linked with data on banks establishments in respondents’ residential and surrounding census tracts from the National Establishment Time Series database, in a series of regression analyses. White older adults have significantly higher rates of banks in their local areas compared with Black and Hispanic older adults, and older adults with higher levels of education have significantly greater local banking access than those with less education. Higher rates of local banking institutions are associated with significantly lower perceptions of neighborhood danger, but not with perceived collective efficacy. This finding emerges when accounting for neighborhood concentrated disadvantage and physical disorder. Local banks may represent neighborhood investment and the broader economic vitality of a community, as well as the ability of communities to meet older adults’ everyday needs in ways that enhance older residents’ feelings of safety. Increasing access to local financial institutions may help attenuate neighborhood-based contributors to inequalities in health and well-being among the aging population.

